# ﻿Two new species of riparian hoppers (Amphipoda, Talitridae) from Trat and Samut Prakan provinces, Thailand

**DOI:** 10.3897/zookeys.1234.140645

**Published:** 2025-04-22

**Authors:** Anotai Suklom, Tosaphol Saetung Keetapithchayakul, Azman Abdul Rahim, Koraon Wongkamhaeng

**Affiliations:** 1 Department of Zoology, Faculty of Science, Kasetsart University, Bangkok 10900, Thailand Kasetsart Univerisy Bangkok Thailand; 2 The Center for Entomology and Parasitology Research; College of Medicine and Pharmacy, Duy Tan University, 120 Hoang Minh Thao, Lien Chieu, Da Nang, Vietnam Duy Tan University Da Nang Vietnam; 3 Marine Ecosystem Research Centre (EKOMAR), Department of Earth Sciences and Environment, Faculty of Science and Technology, Universiti Kebangsaan Malaysia, 43600 UKM Bangi, Selangor, Malaysia Universiti Kebangsaan Malaysia Selangor Malaysia

**Keywords:** *Floresorchestia*, new species, *Platorchestia*, riparian hopper, Talitridae, Thailand

## Abstract

*Floresorchestia* has been recorded from the South African coast throughout the tropical Indo-Pacific and Caribbean seas. *Platorchestia* exhibits a distribution along the coastlines of the Atlantic Ocean and has been documented in the Baltic and Mediterranean seas, North America, Bermuda, and South Africa; however, it has not been recorded in Southeast Asia. This study presents the discovery of two new species of *Floresorchestia* and *Platorchestia* (Crustacea: Amphipoda) from a small creek bank in Trat and Bang Pu, Samut Prakan Province, respectively. These new species, classified as riparian hoppers, significantly contribute to the existing biodiversity in Southeast Asia. *Floresorchestiatrisetosa***sp. nov.** can be distinguished by left mandible lacinia mobilis 4-dentate; gnathopod 2 palm reaching approximately 34%; telson as broad as long, with three robust setae per lobe. *Platorchestiaaquaticus***sp. nov**. can be distinguished by gnathopod 1 subchelate, cuspidactylate, gnathopod 2 palm reaching approximately 35%; telson with three marginal robust setae, and three apical robust setae per lobe.

## ﻿Introduction

The amphipod family Talitridae is diverse and widespread. *Floresorchestia* ranges from warm temperate South Africa across the tropical Indian and Pacific Oceans. *Platorchestia* has been reported on every continent, particularly in temperate zones. These two genera were considered coastal or terrestrial (Bousfield 1984) when all Talitridae were attributed to one of these habitat types and salt marshes. Today, ten distinct ecological hopper types are recognized: marsh, beach, driftwood, sand, field, ground, riparian, forest, moss, and cave (Lowry and Myers 2019). At a generic level, forest hoppers make up the largest group, followed by field hoppers and beach hoppers, but the most specific group is the beach hoppers. *Floresorchestia* is the most ecologically tolerant of the talitrid genera, with members classified as marsh hoppers, beach hoppers, forest hoppers, field hoppers, or riparian hoppers. The wide variety of habitats implies a high adaptation ability, and many studies presumed that the terrestrial species have a coastal *Floresorchestia* ancestor (Bousfield 1984; Lowry and Springthorpe 2015, 2019). *Platorchestia* species, by contrast, are known primarily as beach hoppers, with only one species (*P.negevensis* Myers & Lowry, 2023) previously known as riparian hoppers. In Thailand, there are five species of *Floresorchestia*: *F.boonyanusithii* Wongkamhaeng, Damrongrojwattana & Pattaratumrong, 2016, *F.buraphana* Wongkamhaeng, Damrongrojwattana & Pattaratumrong, 2016, *F.kongsemae* Suklom, Danaisawadi & Wongkamhaeng, 2021, *F.amphawaensis* Suklom, Keetapithchayakul, Abdul Rahim & Wongkamhaeng, 2022 and *F.pongrati* Suklom, Keetapithchayakul, Abdul Rahim & Wongkamhaeng, 2022 (Azman et al. 2014; Wongkamhaeng et al. 2016; Suklom et al. 2021; Suklom et al. 2022). The latter two species were reported in agricultural and urban areas near the Mae Klong River. Although talitrid amphipods can inhabit the terrestrial environment, they require conditions of high humidity and can detect humidity gradients by the virgula davina, the same as terrestrial isopods (Friend and Richardson 1986; Lowry and Springthorpe 2015). Therefore, the dispersal of *Floresorchestia* in Thailand should correspond with the flood plain and may relate to the river basins. The genus *Platorchestia*Bousfield (1982) was included in the subfamily PlatorchestiinaeLowry and Myers (2022). Platorchestiinae is well known for its broad distribution. Currently, *Platorchestia* is present on Atlantic Ocean shores (including the Caribbean, Baltic, Mediterranean, and North seas), the Indian Ocean, the western coast of Australia, the Pacific Ocean, the western US Coast, and the East China Sea (Myers and Lowry 2023). This work is the first record of *Platorchestia* in Southeast Asia and of the first riparian hoppers in the Indo-Pacific. Additionally, the new species of *Platorchestia* are the first records of this genus in Southeast Asia.

## ﻿Materials and methods

**Figure 1.**
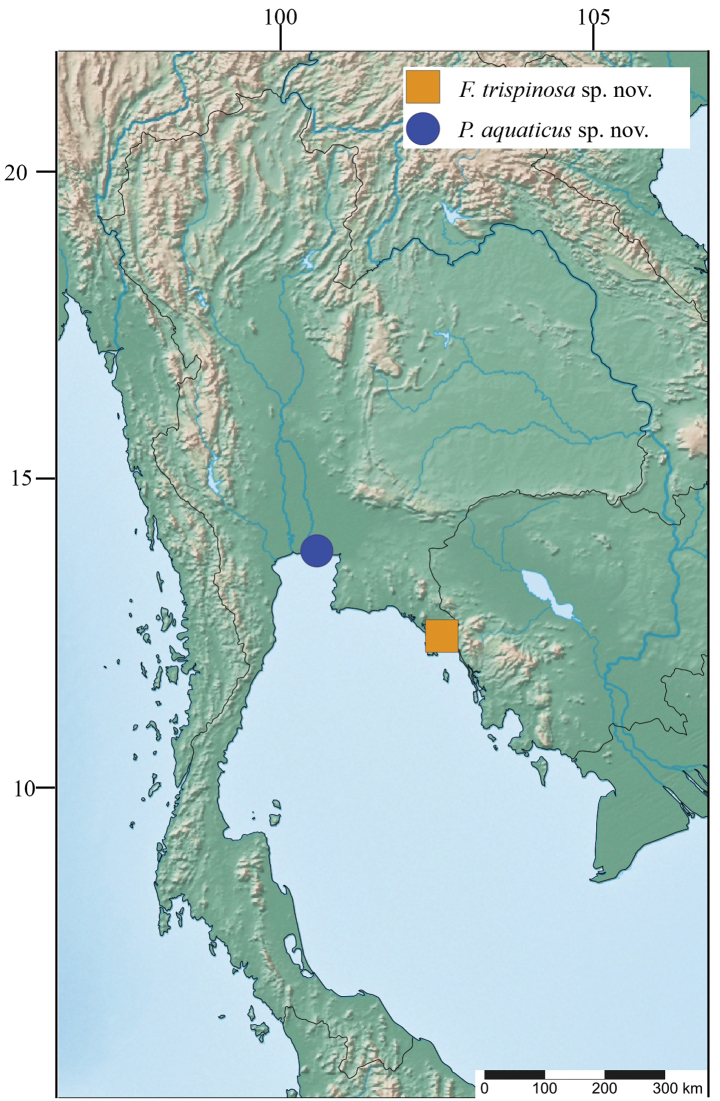
Map showing the sampling area. Orange square represents type locality of *Floresorchestiatrisetosa* sp. nov. and blue circle represents type locality of *Platorchestiaaquaticus* sp. nov.

This study is based on material collected from leaf litter in ponds of rice fields and urban locations in Trat Province, including the shore of Klong Mai and Bang Pu, Samut Prakan, Thailand (Fig. 1). Specimens were collected using a pit-fall trap and were then carefully transferred into plastic containers. They were fixed in 70% ethanol and preserved in 95% ethanol. The specimens were examined under a dissecting microscope and later selected for dissection. Appendages of the specimens were examined, and representative figures were produced using a camera lucida attached to an Olympus CH30 light microscope. Pencil drawings were scanned and digitally inked using an iPad via the Procreate application. Final plates were prepared using Adobe Photoshop CC 2017. Distribution maps were plotted using SimpleMappr (Shorthouse 2010).

**Figure 2.**
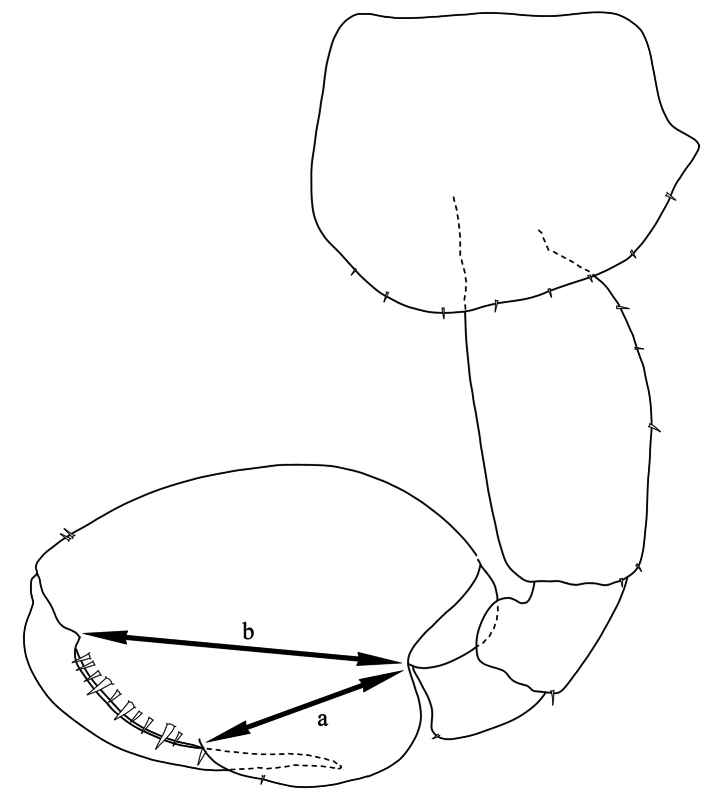
The measurement method for the length of male gnathopod 2 palm and posterior margin of propodus.

The palm length was measured as a percentage of the length of the propodus of male gnathopod 2 and was calculated using the formula 100(1- a/b)% (Fig. 2), where ‘a’ is the length of the posterior margin measured from the seta at the corner of the palm to the base of the propodus and ‘b’ is the length of the propodus measured from the base of the dactylus to the base of the propodus (Lowry and Springthorpe (2015: 6)). Terminology for setae and mouthparts follows Zimmer et al. (2009). Abbreviations used in figures are **A**, antenna; **EP**, epimera; **G**, gnathopod; **LL**, lower lip; **MD**, mandible; **MX**, maxilla; **MP**, maxilliped; **P**, pereopod; **PL** pleopod; **T** telson; **U**, uropod; **UL**, upper lip; **R**, right; **L**, left.

## ﻿Results

### ﻿Systematics

**Superfamily Talitroidea Bulycheva, 1957**

**Family Talitridae Rafinesque, 1815**

**Subfamily Floresorchestiinae Myers and Lowry, 2020**

#### ﻿ Floresorchestia

Taxon classificationAnimaliaAmphipodaTalitridae

Bousfield, 1984

84656FB3-9CF9-5599-A3F4-8FD8FA67E5D2

Orchestia floresiana group: Bousfield 1971: 267.
Floresorchestia Bousfield, 1984: 205. Miyamoto and Morino 2008: 838. Lowry and Springthorpe 2009: 121. Lowry and Springthorpe 2015: 7.

##### Type species.

*Orchestiafloresiana* Weber, 1892, original designation.

##### Diagnostic description.

(modified from Lowry and Springthorpe 2009, 2015) *Antenna 1* short, not longer than article 4 of antenna 2 peduncle. *Antenna 2* peduncular articles slender; article 3 without ventral process. *Left mandible* lacinia mobilis 4–5-cuspidate. *Maxilliped* palp article 2 distomedial lobe well developed, article 4 reduced, button-shaped. *Gnathopod 1* sexually dimorphic; subchelate; posterior margin of merus, carpus and propodus each with lobe covered in palmate setae. *Gnathopod 2* sexually dimorphic; subchelate; dactylus distally attenuated (except *Floresorchestiapapeari* Lowry & Springthorpe, 2015 and *F.ancheidos* (H.K. Barnard, 1916)). *Pereopods* 3–7 cuspidactylate (except *F.odishi* Bhoi, Myers, Kumar & Patro, 2024; pereopods 6 and 7 unidactylate). Pereopods 6 and 7 not sexually dimorphic. *Pleopods* all well developed, biramous. *Epimera* 1–3, 2 and 3, or 2 with slits just above ventral margins, vestigial on epimera 1 (except *F.xueli* Tong, Hoa, Liu, Li & Hou, 2021). *Uropods* 1, 2 not sexually dimorphic. Uropod 1 outer ramus without marginal robust setae. Uropod 2 outer ramus with marginal robust setae. Uropod 3 ramus subequal in length to peduncle. *Telson* with 3–7 robust setae.

**Female** (sexually dimorphic characters). *Gnathopod 1* posterior margin of merus, carpus and propodus each without lobe covered in palmate setae. *Gnathopod 2* mitten-shaped. *Oostegites* on gnathopod 2 to pereopod 5; setae straight.

##### Species composition.

*Floresorchestia* includes 29 species: *F.amphawaensis* Suklom, Keetapithchayakul, Abdul Rahim & Wongkamhaeng, 2022; *F.andrevo* Lowry & Springthorpe, 2015; *F.anomala* (Chevreux, 1901); *F.anoquesana* (Bousfield, 1971); *F.anpingensis* Miyamoto & Morino, 2008; *F.boonyanusithii* Wongkamhaeng, Dumrongrojwattana & Pattaratumrong, 2016; *F.buraphana* Wongkamhaeng, Dumrongrojwattana & Pattaratumrong, 2016; *F.floresiana* (Weber, 1892); *F.hanoiensis* Hou & Li, 2003; *F.kalili* Lowry & Springthorpe, 2015; *F.kongsemae* Suklom, Danaisawadi & Wongkamhaeng, 2021; *F.laurenae* Lowry & Springthorpe, 2015; *F.malayensis* (Tattersall, 1922); *F.mkomani* Bichang’a & Hou in Bichang’A et al., 2021; *F.odishi* Bhoi, Myers, Kumar & Patro, 2024; *F.oluanpi* Lowry & Springthorpe, 2015; *F.palau* Lowry & Myers, 2013; *F.papeari* Lowry & Springthorpe, 2015; *F.pohnpei* Lowry & Myers, 2013; *F.poorei* Lowry & Springthorpe, 2009; *F.pongrati* Suklom, Keetapithchayakul, Abdul Rahim & Wongkamhaeng, 2022; *F.samoana* (Bousfield, 1971); *F.seringat* Lowry & Springthorpe, 2015; *F.thienemanni* (Schellenberg, 1931); *F.trisetosa* sp. nov.; *F.vitilevana* (J.L. Barnard, 1960); *F.xueli* Tong, Hao, Liu, Li & Hou, 2021; *F.yap* Lowry & Springthorpe, 2015; *F.yehyuensis* Miyamoto & Morino, 2008.

##### Remarks.

The subfamily Floresorchestiinae is comprised of three genera (*Austropacifica*, *Floresorchestia*, and *Gazia*) and is defined by vertical slits on the ventral margin of epimera 1–3, 2 and 3, or only 2. *Floresorchestia* differs from *Gazia* in having a palmate lobe on the merus carpus and propodus of male gnathopod 1, whereas *Gazia* lacks a palmate lobe on the merus of male gnathopod 1. *Floresorchestia* differs from *Austropacifica* in not having the mid-medial robust setae with a modified tip on the outer ramus of uropod 1.

#### ﻿ Floresorchestia trisetosa sp. nov.

Taxon classificationAnimaliaAmphipodaTalitridae

8745F25B-0E46-54F6-A899-0C4E118215D6

https://zoobank.org/5C7AA8E3-B49C-4264-80B0-0DBAC71E3FEF

Figs 3 , 4 , 5 , 6

##### Type material.

***Holotype*** • Thailand, 1 ♂; Muang Trat District, Trat; 12°15'12.9"N, 102°30'31.3"E; 21 February 2021; Anotai Suklom; pit fall trap; THNHM-lv-20866. ***Allotype*** • 1 ♀; collected with holotype; THNHM-lv-20867. ***Paratypes*** • 2 ♂ and 2 ♀ collected with holotype; THNHM-lv-20868.

##### Type locality.

A small creek near the restaurant, Muang Trat District, Trat, Thailand.

##### Ecological type.

Riparian hoppers (edges of lakes under stones or in very wet vegetation, near or in streams, rivers, creeks, cascades, and waterfalls).

##### Diagnosis.

Mandible lacinia mobilis 4-dentate. Gnathopod 1 with palmate lobes on merus, carpus and propodus; palm acute. Gnathopod 2 propodus palm reaching ~ 33% along posterior margin; dactylus attenuated distally. Pereopod 4 dactylus thickened proximally, with slight notch midway along posterior margin. Epimeron 2 and 3 with slits just above ventral margins. Uropod 1 outer ramus without marginal robust seta, with three marginal robust setae in one row. Uropod 3 peduncle with one robust seta; ramus with two apical robust setae. Telson with one apical robust seta, and two lateral robust seta per lobe.

##### Description.

Based on male holotype 8.7 mm, THNHM-lv-20866.

**Figure 3.**
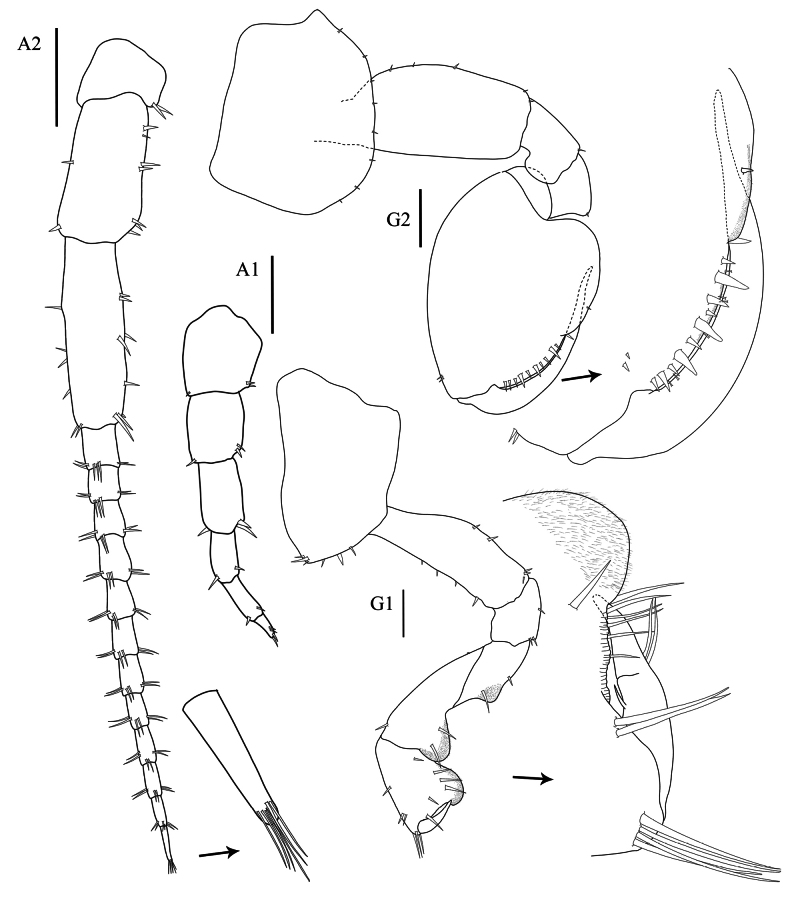
*Floresorchestiatrisetosa* sp. nov., holotype, male, 5.5 mm, THNHM-Iv-20866. Scale bars: 0.5 mm (**A2, G1, G2**); 0.2 mm (**A1**).

**Figure 4.**
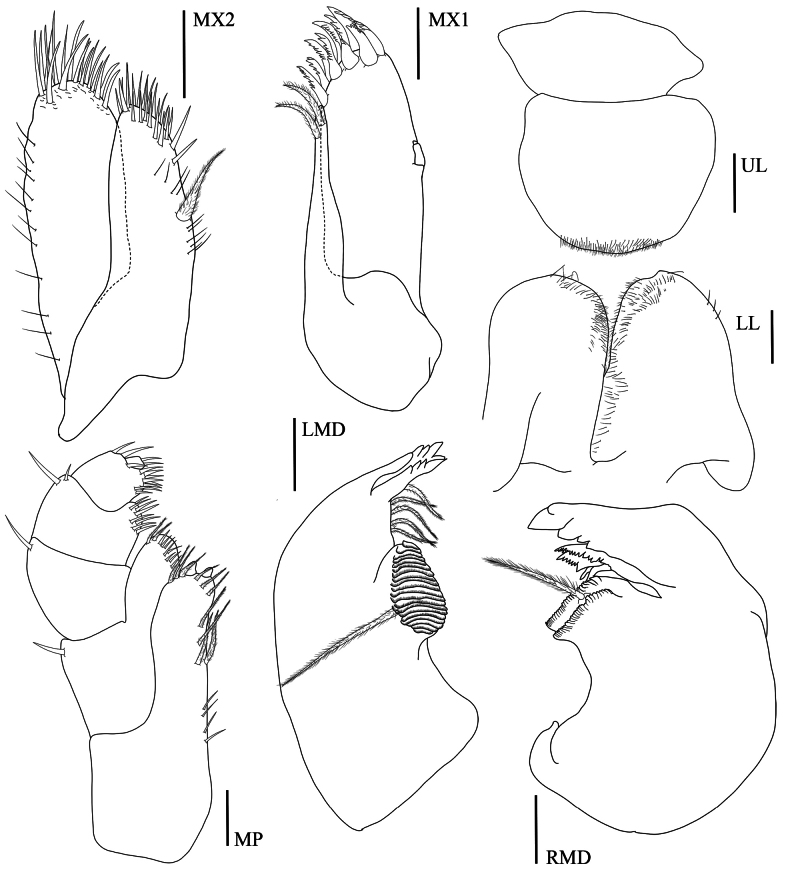
*Floresorchestiatrisetosa* sp. nov., holotype, male, 5.5 mm, THNHM-Iv-20866. Scale bars: 0.2 mm (**X1, MX2, MP, LMD, RMD**); 0.1 mm (**UL, LL**).

**Head. *Eye*** large (> 1/3 head length). ***Antenna 1*** (Fig. 3, A1) short, rarely longer than article 4 of antenna 2 peduncle. ***Antenna 2*** (Fig. 3, A2) < 1/2 body length, peduncular articles slender, article 5 longer than article 4. ***Upper lip*** (Fig. 4, UL) without robust setae, broad, rounded apex, apical marginal with fine setule. ***Lower lip*** (Fig. 4, LL) without inner plate, with fine setule on apex and inner margin. ***Left mandible*** (Fig. 4, LMD) incisor 5-dentate; left lacinia mobilis 4-dentate and five pappose setae type I in one row; molar strong with 20 striations and one pappose setae type II on the distal of molar. ***Right mandible*** (Fig. 4, RMD) incisor 5-dentate; lacinia mobilis with numerous cusps; molar strong with 26 striations and 1 distal pappose seta. ***Maxilla 1*** (Fig. 4, MX1) inner plate slender with 2 apical papposerrate setae type I; outer plate with 9 robust serrate setae type I and small palp 2-articulate on outer lateral margin. ***Maxilla 2*** (Fig. 4, MX2) inner plate slightly shorter than outer plate, subapical margin with 19 robust setae, with one mediolateral papposerrate seta type I: outer plate with 18 apical robust setae in two rows. ***Maxilliped*** (Fig. 4, MP) inner plate apical and subapical margins with papposerrate setae type II, and two large conical robust setae; outer plate subapical margin with robust setae and two pappose setae; palp article 2 distomedial lobe well developed; article 4 reduced, button shaped.

**Pereon. *Gnathopod 1*** (Fig. 3, G1) sexually dimorphic; subchelate; coxa smaller than coxa 2, anterior margin slightly convex distally; basis slightly expanded posteriorly, anterior margin with four robust setae, posterior margin with two marginal robust setae and two terminal robust setae; ischium subquadrate, shortest, anterior lobe slightly rounded; posterior margin of merus, carpus and propodus each with lobe covered in palmate setae; carpus longer than propodus, carpus 1.27× as long as propodus, carpus 1.9× as long as broad; propodus ‘subtriangular’ with well-developed posterodistal lobe, anterior margin with three groups of robust setae, lateral surface with four cuspidate setae, posterolateral surface with three serrate setae, medial surface with five cuspidate setae, posterior margin without cuspidate setae, with three serrate setae; palm transverse, with five serrate setae; dactylus longer than palm, without spine patch on posterodistal corner . ***Gnathopod 2*** (Fig. 3, G2) sexually dimorphic; subchelate; coxal gill simple (or slightly lobate); basis slightly expanded; ischium with distally rounded posterodistal lobe on medial surface; posterior margin of merus, carpus and propodus each without lobe covered in palmate setae; carpus reduced, enclosed by merus and propodus, posterior absent, not projecting between merus and propodus; propodus subovate, 1.4× as long as wide; palm acute, reaching ~ 33% along posterior margin, smooth, evenly rounded, lined with robust setae, posteromedial surface of propodus with groove, without cuticular patch at corner of palm; dactylus longer than palm, without anteroproximal bump, posterior margin smooth, attenuate distally; gill lobate.

**Figure 5.**
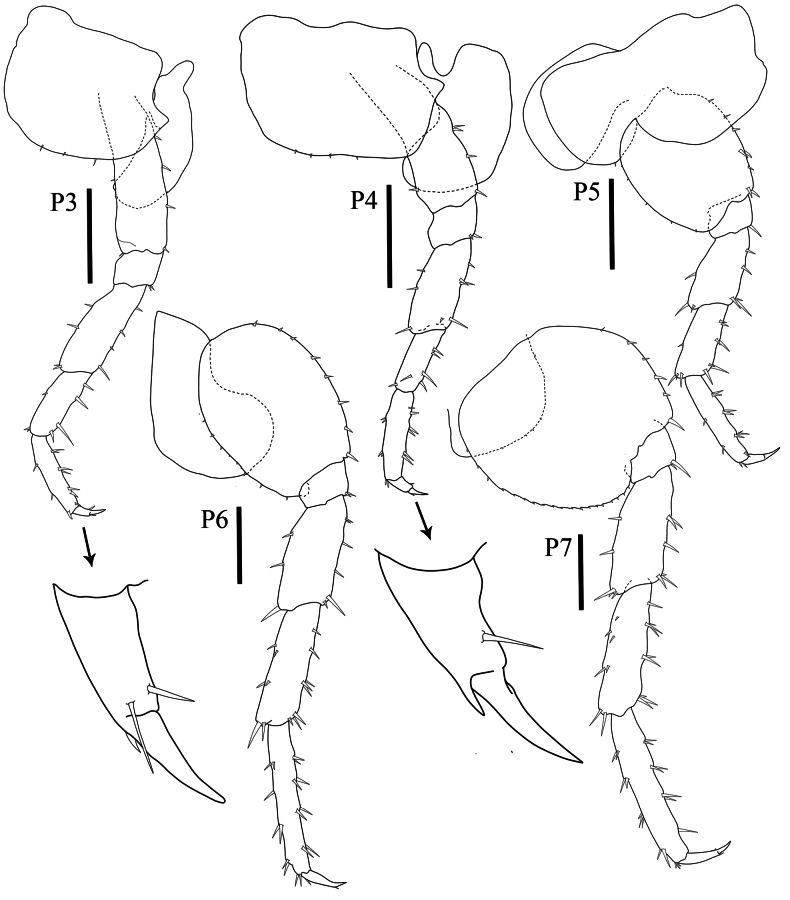
*Floresorchestiatrisetosa* sp. nov., holotype, male, 5.5 mm, THNHM-Iv-20866. Scale bars: 0.5 mm.

***Pereopods 3–4*** (Fig. 5, P3–P7) coxae wider than deep. ***Pereopods 3–7*** cuspidactylate; dactyli without anterodistal patch of many rows of tiny setae. ***Pereopod 3*** (Fig. 5, P3) coxa wider than deep, ventral margin with four robust setae, posterior margin with acute process; basis slightly expanded, anterior margin slightly straight and naked, posterior margin with four robust setae; ischium subrectangular, shortest; merus longer than carpus and propodus, distally expanded, anterior margin with four robust setae, posterior margin with four robust setae; carpus as long as propodus, anterior margin with three setae, posterior margin with four robust setae; propodus slender, anterior margin with three robust setae, posterior margin with eight robust setae; dactylus without notch on posterior margin, anterior corner with one robust seta and posterior inner view with two robust setae. ***Pereopod 4*** (Fig. 5, P4) significantly shorter than pereopod 3; coxa wider than deep, posterior margin with acute process; basis slightly expanded distally, anterior margin distally convex, posterior margin with five robust setae; ischium shortest, subquadrate, distally convex; merus longer than carpus and propodus; carpus significantly shorter than carpus of pereopod 3; propodus slender, anterior margin with three groups of robust setae, posterior margin with three groups of robust setae; dactylus thickened proximally with a notch midway along posterior margin, dactylus without anterodistal setal patch. ***Pereopod 5*** (Fig. 5, P5) coxa bilobed, anterior lobe distinctly larger than posterior lobe, posterior lobe with setae on ventral margin; basis ovate, anterior margin with eight robust setae, posterior margin with five minute setae; ischium shortest, subrectangular, posterior margin distally convex; merus as long as carpus, distally expanded, anterior margin with five robust setae; carpus shorter than propodus, anterior margin with seven robust setae, posterior margin with one marginal robust seta and three robust setae distally; propodus distinctly longer than carpus; dactylus without anterodistal setal patch. ***Pereopods 6–7*** (Fig. 5, P6 and P7) not sexually dimorphic. ***Pereopod 6*** (Fig. 5, P6) subequal in length to pereopod 7; coxa posterior lobe larger than anterior lobe, inner view posteroventral corner rounded, posterior margin perpendicular to ventral margin, posterior lobe without ridge, without marginal setae; basis ovate, anterior margin with eight robust setae, posterior margin with nine or ten minute setae; ischium shortest; merus slightly expanded, anterior margin crenulate with six robust setae, posterior margin convex with three robust setae; carpus as long as merus, anterior margin crenulate with eight robust setae, posterior margin with six robust setae in three groups; propodus longer than carpus, slender, anterior margin with eight robust setae in four groups, posterior margin with nine robust setae; dactylus slender with setae in posterior corner. ***Pereopod 7*** (Fig. 5, P7) coxa reduced; basis sub ovate, lateral sulcus absent, posterior margin with distinct minute serrations, each with a small seta, posterodistal lobe present, shallow, broadly rounded; ischium shortest, subrectangular with posterior rounded process; merus posterior margin expanded distally, subtriangular; propodus slightly longer than carpus, anterior margin with seven robust setae, posterior margin with eight robust setae and three terminal minute setae; dactylus slender and with subapical setae.

**Figure 6.**
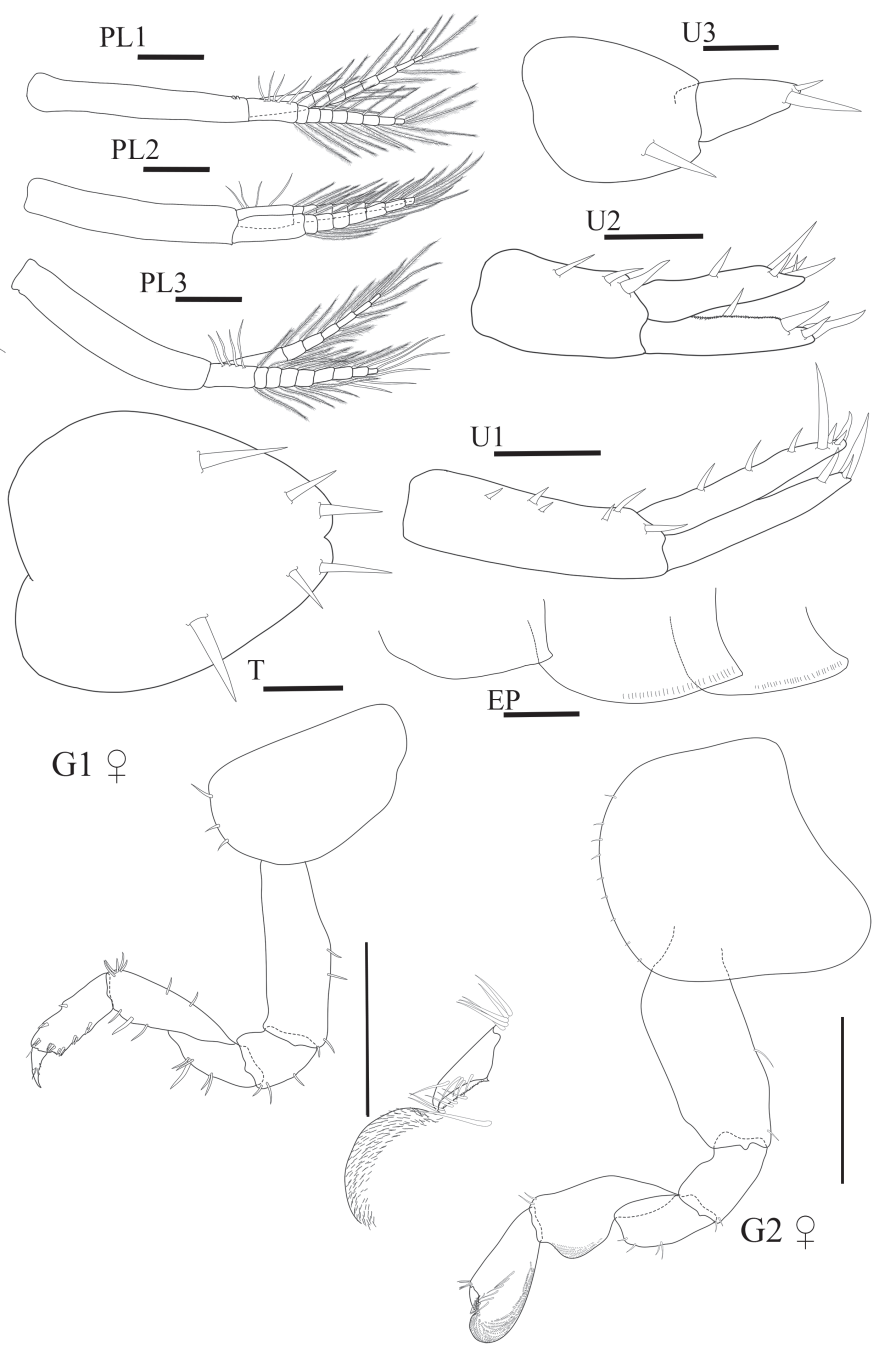
*Floresorchestiatrisetosa* sp. nov., holotype, male, 5.5 mm, THNHM-Iv- 20866, allotype, female, 4.7 mm, THNHM-Iv-20867. Scale bars: 0.25 mm (**PL1–3, EP**); 0.2 mm (**U1–2, U3**); 0.1 mm (**T**) 0.5 mm (**G1♀, G2** ♀).

**Pleon. *Pleopods 1–3*** well developed, biramous; peduncle ventral margin without robust setae; rami without ventral robust setae. ***Pleopod 1*** (Fig. 6, PL1) peduncle longer than rami; inner ramus subequal in length to outer ramus, with eight articles; outer ramus with nine articles. ***Pleopod 2*** (Fig. 6, PL2) peduncle longer than rami; inner ramus with eight robust setae; outer ramus with eight robust setae. ***Pleopod 3*** (Fig. 6, PL3) peduncle longer than rami; inner ramus with nine robust setae; outer ramus with seven robust setae. ***Epimeron 1*** (Fig. 6, EP) posterior corner slightly projecting, and without slits. ***Epimera 2 and 3*** each with slits above ventral margin; posterior ventral corner and ventral margin smooth. ***Uropod 1*** (Fig. 6, U1) peduncle slightly longer than ramus with 6 robust setae, distolateral robust seta present, small (< 1/4 length of outer ramus), with simple tip; inner ramus subequal in length to outer ramus, inner ramus with three marginal robust setae (1 row), with four apical robust setae; outer ramus without marginal robust setae. ***Uropod 2*** (Fig. 6, U2) not sexually dimorphic; peduncle shorter than rami with 3 robust setae; inner ramus subequal in length to outer ramus, with marginal robust setae, with one lateral robust seta; outer ramus with marginal robust setae in one row. ***Uropod 3*** (Fig. 6, U3) peduncle longer than ramus with one robust seta; ramus not fused to peduncle; ramus 1.6× as long as broad. ***Telson*** (Fig. 6, T) longer than broad, apically incised, dorsal midline less than halfway, with one apical robust seta, and two lateral robust setae.

**Female** (Fig. 6) (sexually dimorphic characters). Based on allotype, female 4.7 mm. THNHM-lv-20867.

**Pereon. *Gnathopod 1*** (Fig. 6, G1♀) parachelate; coxa anterior margin straight, anteroventral margin with three robust setae; basis slender, anterior margin straight, posterior margin slightly expanded distally with two subterminal robust setae; ischium shortest, subrectangular, anterior margin slightly convex; posterior margin of merus, carpus and propodus without lobe covered in palmate setae; merus triangular with two groups of robust setae on posterior margin; carpus subtriangular, anterior margin curved with four terminal setae, posterior margin slightly convex with three robust setae; propodus slender, anterior margin crenulate with two marginal robust setae and four terminal setae, posterior margin crenulate with three lateromedial robust setae; dactylus slender, anterior corner with one robust seta, posterior margin with one seta and two lateromedial robust setae. ***Gnathopod 2*** (Fig. 6, G2♀) mitten-shaped; coxa as long as deep, anterior margin straight, posterior margin straight, ventral margin with minute setae; basis expanded, anterior margin naked, posterior margin straight with one seta; ischium as long as merus, anterior margin with rounded lobe, posterior margin straight with one terminal seta; merus shorter than carpus, anterior margin with rounded lobe, posterior margin with rounded lobe; carpus and propodus each with lobe covered in palmate setae; carpus subtriangular, longer than propodus; propodus subovate, lateromedial corner with three robust setae; dactylus slender, shorter than palm, anterior margin with one robust seta, posterior margin with two robust setae.

##### Distribution.

Thailand. Trat town Municipality, Trat.

##### Etymology.

Named for the character of three robust setae on each telsonic lobe.

##### Remarks.

*Floresorchestiatrisetosa* sp. nov. is closely related to *F.boonyanusithii* and *F.amphawaensis* by having the gnathopod 2 palm reaching between 31–35%, dactylus posterior margin smooth, proximal tooth absent, and mandible left lacinia mobilis 4-dentate. *F.trisetosa* can be separated from those two species by the combination of characteristics as follows (other species in paratheses): (1) gnathopod 1 carpus 1.2 × propodus (1.4× in *F.boonyanusithii*, 1.5× in *F.amphawaensis*); (2) uropod 1 peduncle with six robust setae (6 in *F.boonyanusithii*; 4 in *F.amphawaensis*); (3) uropod 3 peduncle with two robust setae (4 in *F.amphawaensis* and 2 in *F.boonyanusithii*); (4) telson longer than broad, telsonic lobe with three robust setae (vs 4 robust setae in *F.boonyanusithii*, and *F.amphawaensis*).

**Figure 7.**
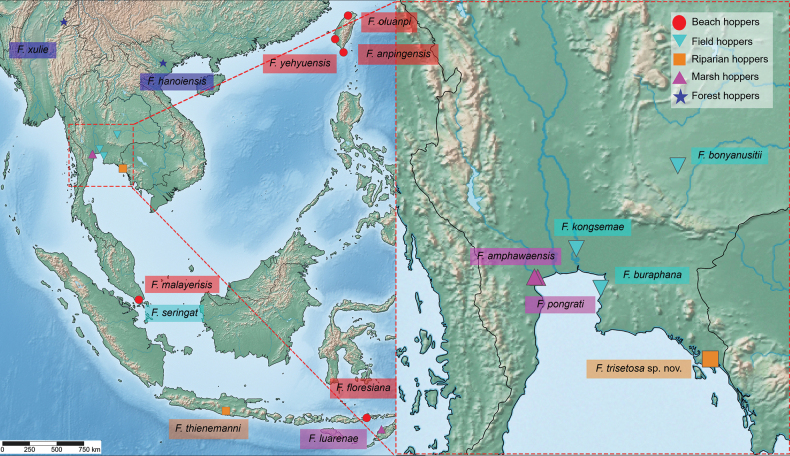
Ecological groups of *Floresorchestia* species in Southeast Asia.

*Floresochestiatrisetosa* is the first riparian hopper to be reported from Thailand (Fig. 7).

###### ﻿Subfamily Platorchestiinae Lowry & Myers, 2022

#### ﻿ Platorchestia

Taxon classificationAnimaliaAmphipodaTalitridae

Bousfield, 1982

E620BDBD-67E2-5CBA-99F6-A0D4BE772DB8

Platorchestia Bousfield, 1982: 26; Jo 1988: 160; Richardson 1991: 186; Morino and Ortal 1995: 825; Miyamoto and Morino 2004: 68; Myers and Lowry 2023: 486.

##### Type species.

*Platorchestiaplatensis* (Krøyer, 1845), original designation.

##### Diagnostic description.

(modified from Myers and Lowry 2023) ***Antenna 1*** short, not longer than article 4 of antenna 2 peduncle. ***Antenna 2*** peduncular article 3 without ventral plate; articles 4 and 5 occasionally incrassate in males. ***Maxilliped*** palp article 2 with distomedial lobe; article 4 reduced, button-shaped. ***Gnathopod 1*** sexually dimorphic; subchelate; posterior margin of carpus and propodus each lobe covered in palmate setae; dactylus cuspidactylate. ***Gnathopod 2*** subchelate in male, mitten-shaped in female; basis narrow or slightly expanded; propodus palm posterodistal corner without protuberance. ***Pereopods 3–7*** cuspidactylate. Pereopod 7 occasionally incrassate in male. ***Uropod 1*** outer ramus without marginal robust setae. ***Telson*** with apical and marginal robust setae.

##### Species composition.

*Platorchestia* contains 13 species: *P.ano* Lowry & Bopiah, 2013; *P.aquaticus* sp. nov.; *P.crassicornis* (Costa, 1867); *P.exter* Myers & Lowry, 2023; *P.griffithsi* Myers & Lowry, 2023; *P.munmui* Jo, 1988; *P.negevensis* Myers & Lowry, 2023; *P.oliveirae* Myers & Lowry, 2023; *P.pachypus* (Derzhavin, 1937); *P.pacifica* Miyamoto & Morino, 2004; *P.paraplatensis* Serejo & Lowry, 2008; *P.platensis* (Krøyer, 1845); *P.smithi* Lowry, 2012.

##### Remarks.

Miyamoto and Morino (2004) established three groups of *Platorchestia* based on the presence or absence of sexual dimorphism. Lowry and Myers (2022) established the new subfamily Platorchestiinae, which accommodates 15 genera, including *Platorchestia*. The *Platorchestia* sensu stricto is equivalent to Group 1 of Miyamoto (antenna 2 and pereopods 6 and 7 are strongly sexually dimorphic and represented by supralittoral species). Subsequently, Myers and Lowry (2023) described three new *Platorchestia* species and provided the diagnostic characteristics as antenna 2 sexually dimorphic by articles 4 and 5, which are generally incrassate in males and pereopod 7 often incrassate in male articles 5–7. The combination of antenna 2 and pereopod 7 incrassate was classified into Group 1 and Group 2 by Miyamoto and Morino (2004).

#### ﻿ Platorchestia aquaticus sp. nov.

Taxon classificationAnimaliaAmphipodaTalitridae

E37C7474-080E-5EAA-B81B-C594F65D4612

https://zoobank.org/C7E46D2E-806F-48E8-ACBC-EB8F6322C004

**Figure 8.**
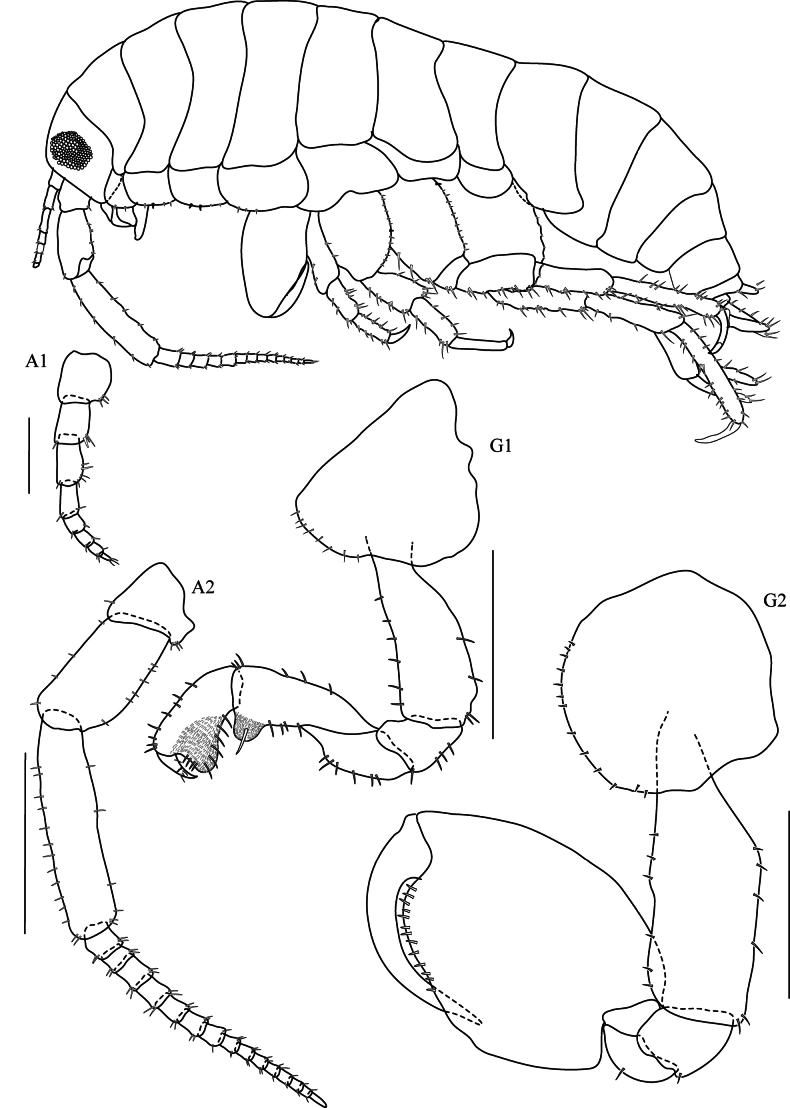
*Platorchestiaaquaticus* sp. nov., holotype, male, 8.7 mm, THNHM-Iv- 20869. Scale bars: 0.2 mm.

**Figure 9.**
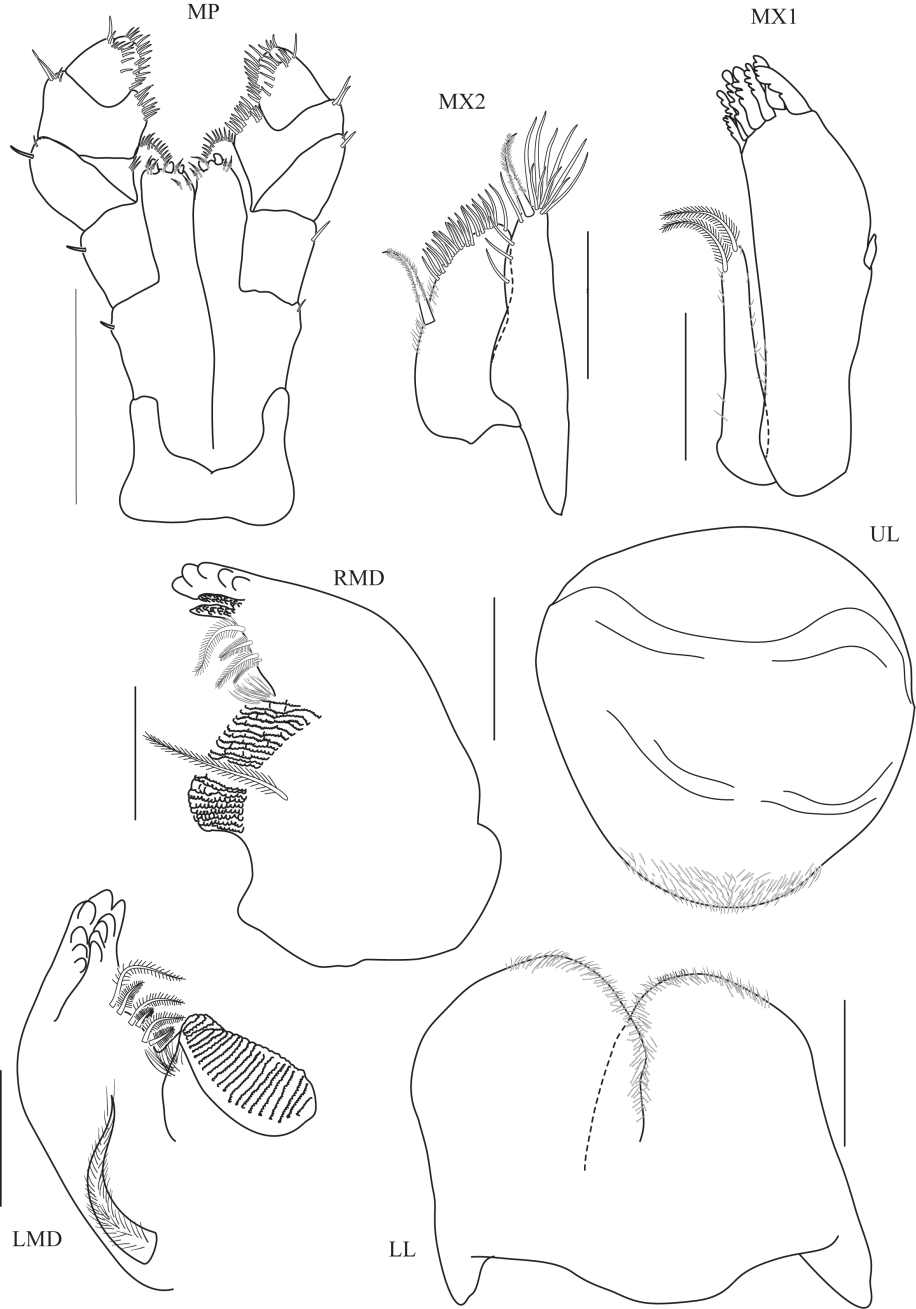
*Platorchestiaaquaticus* sp. nov., holotype, male, 8.7 mm, THNHM-Iv- 20869. Scale bars 0.2 mm.

**Figure 10.**
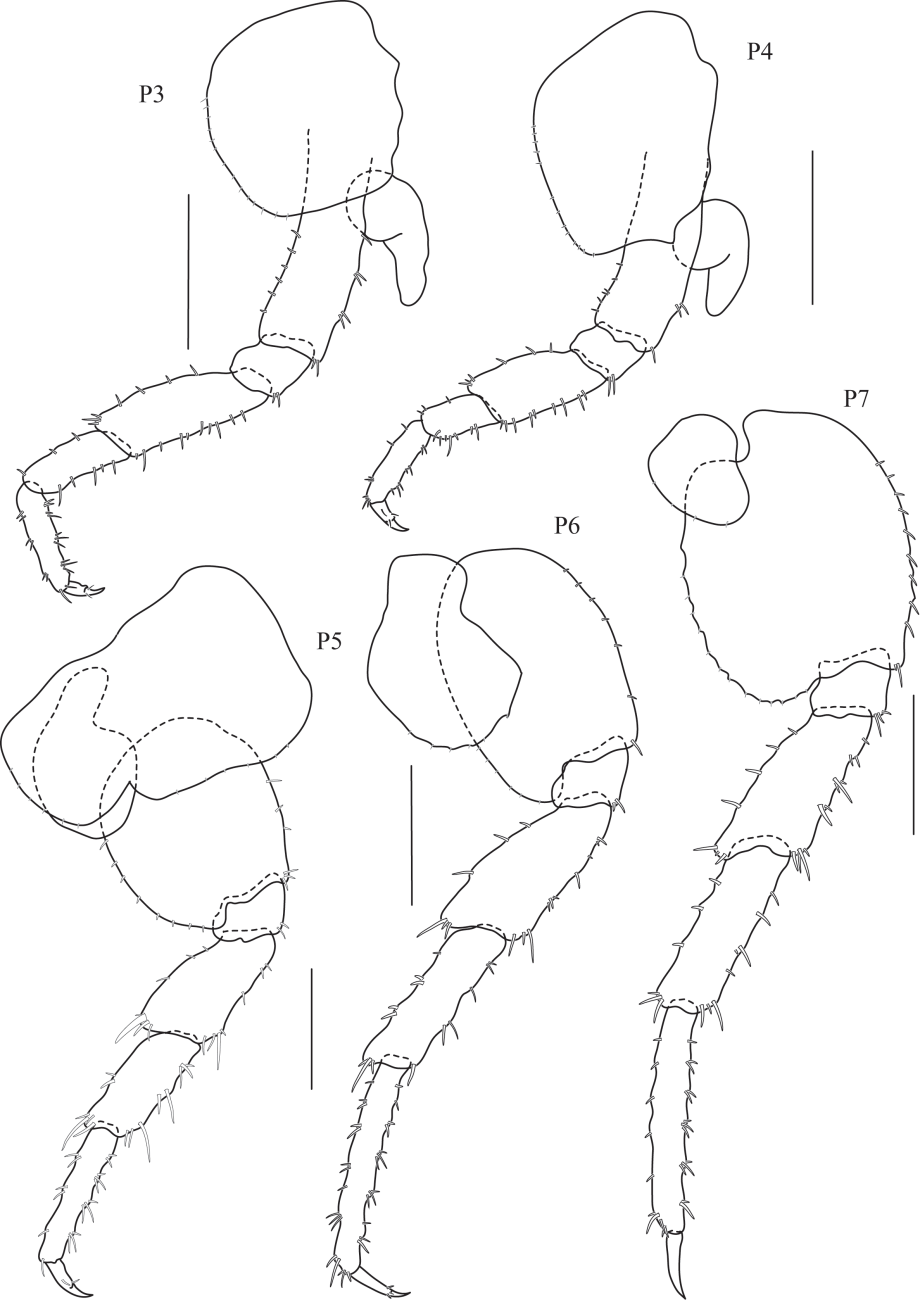
*Platorchestiaaquaticus* sp. nov., holotype, male, 8.7 mm, THNHM-Iv- 20869.Scale bars: 0.5 mm.

**Figure 11.**
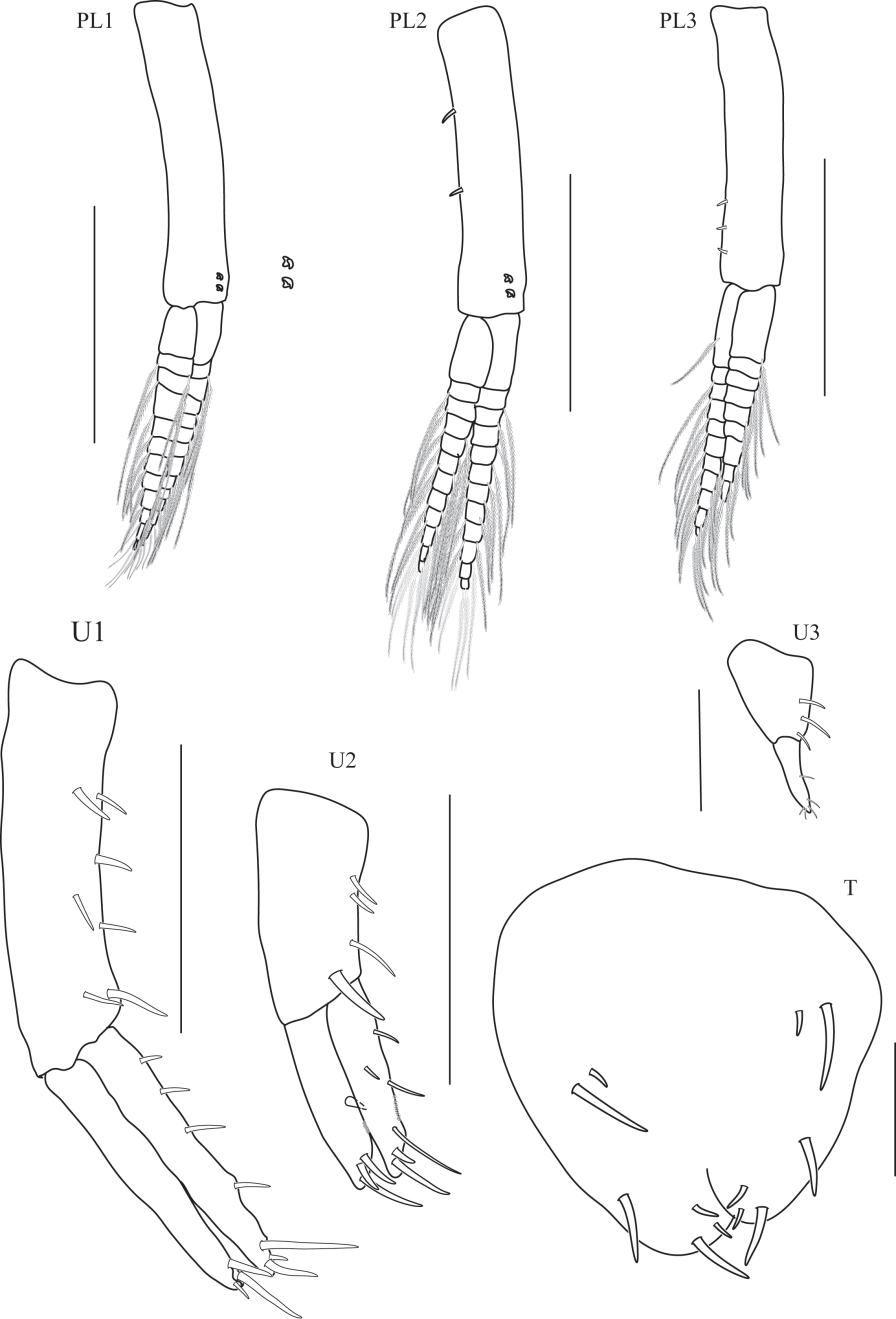
*Platorchestiaaquaticus* sp. nov., holotype, male, 8.7 mm, THNHM-Iv- 20869. Scale bars: 0.5 mm (**PL1–3**); 0.2 mm (**U1–2**); 0.1 mm (**U3, T**).

**Figure 12.**
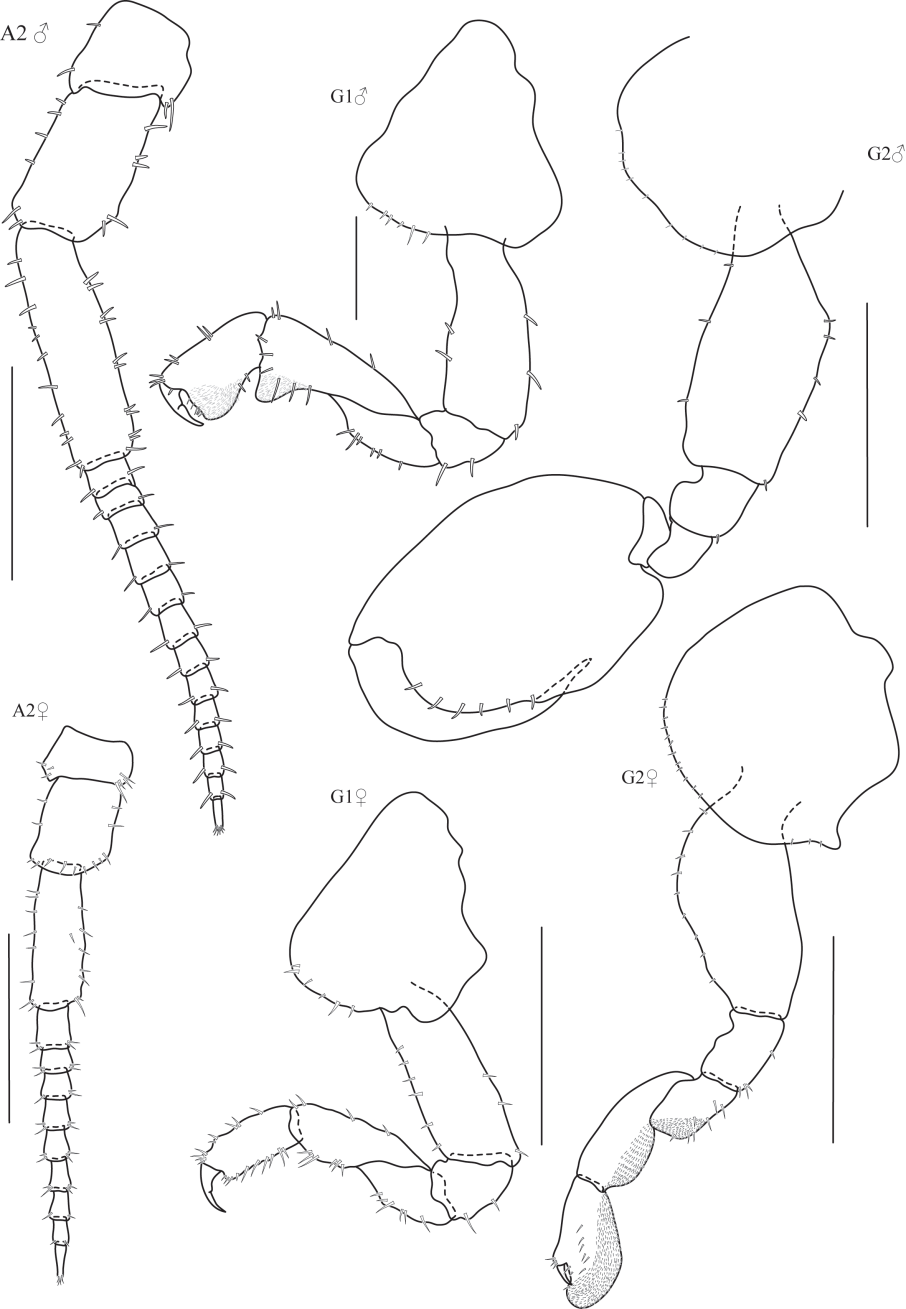
*Platorchestiaaquaticus* sp. nov., paratype male 7.9 mm, THNHM-Iv- 20871, And allotype female 7.8 mm, THNHM-Iv- 20870. Scale bars: 0.5 mm (**A2, G2**); 0.2 mm (**G1**).

Figs 8 , 9 , 10 , 11 , 12

##### Type material.

***Holotype*** • Thailand, 1 ♂; Bang Pu, Samut Prakan; 13°31.73'N, 100°38.17'E; 21 February 2021; Anotai Suklom; pit fall trap; THNHM-lv-20869. ***Allotype*** • 1 ♀; collected with holotype; THNHM-lv-20870. ***Paratypes*** • 2 ♂ and 2 ♀ collected with holotype; THNHM-lv-20871.

##### Type locality.

On the shore of Klong Mai, Bang Pu, Samut Prakan, Thailand.

##### Habitat.

Riparian hoppers, living near urban areas in Bang Pu, Samut Prakan.

##### Ecological type.

Riparian hoppers (on the shore of canal under leaf litter or around the fibrous root of aquatic plants).

##### Diagnosis.

Male antenna 2 and pereopod 7 strongly sexually dimorphic. Male gnathopod 1 rudimentary cusp on dactylus. Male gnathopod 2 propodus without notch on palmar margin. Coxa 6 posterior lobe with anterodistal corner subquadrate, with process, one or two marginal setae, posterior margin perpendicular to ventral margin, outer surface with ridge. Pleopod 2 with two marginal robust setae; pleopod 3 with three marginal robust setae. Uropod 1 peduncle with nine or ten robust setae in two rows. Uropod 3 ramus with one marginal robust seta.

##### Description.

Based on holotype, male, 8.72 mm, THNHM-lv-20869. **Head. *Eye*** medium ~ 1/3–1/5 of head length. ***Antenna 1*** (Fig. 8, A1) short, slightly longer than article 4 of antenna 2. ***Antenna 2*** (Fig. 8, A2) slightly incrassate, shorter than half body length; peduncular articles occasionally expanded, with small setae along the peduncle; article 4 shorter than article 5; flagellum with 15 articles, final article cone-shaped with apical cluster of setae. ***Upper lip*** (Fig. 9, UL) broad, deep, apex round, without robust setae, apical margin with fine setules. ***Lower lip*** (Fig. 9, LL) present; without inner plate, with fine setules on the apex and inner margins. ***Left mandible*** (Fig. 9, LMD) incisor 5-dentate; lacinia mobilis 5-dentate, with six pappose setae type I in one row; molar strong and concave, with 22 striations, with cluster of fine setae on anterior side and one pappose setae type II on the dorsal side of molar. ***Right mandible*** (Fig. 9, RMD) incisor 5-dentate; lacinia mobilis with numerous cusps and four pappose setae type I in one row; molar strong and convex, with 22 striations, with cluster of fine setae on anterior side and one pappose seta type II on the dorsal side. ***Maxilla 1*** (Fig. 9, MX1) inner plate slender with two apical papposerrate setae type I; outer plate with seven robust serrate setae type I; outer margin with small 2-articulate palp. ***Maxilla 2*** (Fig. 9, MX2) inner plate slightly shorter than outer plate; with 25 subapical setae, one papposerrate seta type I and 15 simple slender setae on inner margin; outer plate with 12 simple setae type I and one papposerrate seta type I, inner margin with four robust setae. ***Maxilliped*** (Fig. 9, MP) inner plate with apical papposerrate setae type II and three large conical robust setae; outer plate with apical papposerrate setae; palp article two distomedial lobe well developed with numerous simple setae; article 4 present, reduced.

**Pereon. *Gnathopod 1*** (Fig. 8, G1) sexually dimorphic; subchelate, coxa smaller than coxa 2, ventral margin with eight robust setae, anterior margin straight; basis expanded posteriorly, anterior margin with six robust setae, posterior margin with four robust setae; posterior margin of carpus and propodus each with lobe covered in palmate setae; ischium shortest; merus and carpus triangular; carpus 1.3× longer than propodus; propodus subtriangular with well-developed posterior lobe, palm straight with four robust setae in one row; dactylus cuspidactylate, shorter than palm. ***Gnathopod 2*** (Fig. 8, G2) sexually dimorphic; subchelate; coxa as wide as deep without posterior process, ventral margin convex with 13 robust setae; basis expanded, anterior margin straight with six robust setae, posterior margin slightly concave with six robust setae; ischium subrectangular, anterior margin without notch; merus subequal in length to carpus, convex on posterior margin; carpus triangular; reduce; enclosed by merus and propodus, posterior lobe absent; propodus subovate, palm acute and reaching 34.5–35% along posterior margin, 1.5× as long as wide, posteromedial surface of propodus with groove, anterior margin with one distal robust seta, palmar margin slightly convex with 14 robust setae; dactylus longer than palm and fitting in facial groove of propodus, attenuated distally. ***Pereopod 3*** (Fig. 10, P3) coxa wider than deep without posterior process, ventral margin slightly convex and with 15 robust setae, anterior margin straight, anterior and posterior margins naked; basis slightly expanded distally, anterior margin straight with six robust setae, posterior margin slightly crenulate, with four groups of robust setae; ischium shortest, anterior margin with rounded process; merus slightly expanded, longer than carpus and propodus, anterior margin slightly rough and with seven robust setae, posterior margin slightly straight and with ten robust setae; carpus as long as propodus, anterior margin with three robust setae, posterior margin with five robust setae; propodus anterior margin with three groups of two robust setae in each group, posterior margin with three groups of three robust setae in each group, with two distal robust setae; dactylus posterior margin with one seta on anterior margin. ***Pereopod 4*** (Fig. 10, P4) similar to pereopod 3 but shorter; coxa subrectangular, wider than deep, posterior margin with posterior process, ventral margin with 13 robust setae; basis slightly expanded distally, anterior margin with group of distal robust setae; ischium shortest, anterior margin convex, posterior margin straight with two robust setae; merus slightly expanded, longer than carpus and propodus, anterior margin crenulate with six robust setae, posterior margin crenulate with four groups of robust setae with two robust setae in each group; carpus as long as propodus, anterior margin straight with three robust setae, posterior margin slightly crenulate with seven robust setae; propodus slender, anterior margin with seven robust setae, posterior margin with three groups of robust setae; dactylus slender and longer than pereopod 3, thickened proximally with notch along posterior margin. ***Pereopod 5*** (Fig. 10, P5) coxa bilobed, anterior lobe distinctly larger than posterior lobe, ventral margin with minute setae; basis ovate, anterior margin with seven robust setae, posterior margin with eight minute setae; ischium subrectangular, shortest, posterior margin notched; merus expanded distally, subequal in length to carpus, anterior margin slightly concave with seven robust setae, posterior margin convex with seven robust setae; carpus anterior margin crenulate with three groups of robust setae with three robust setae in each group, posterior margin slightly straight; propodus slender, longer than merus and carpus, anterior margin with four groups of robust setae with three robust setae in each group, posterior margin crenulate with five robust setae; dactylus with two robust setae on anterior margin. ***Pereopod 6*** (Fig. 10, P6) coxa bilobed, anterior lobe very small, anterior margin straight, posterior lobe inner view posteroventral corner rounded, posterior margin perpendicular to ventral margin, ventral margin serrate and with minute setae; basis ovate, anterior margin with eight robust setae, posterior margin with six minute setae; ischium shortest; anterior margin straight, posterior margin with rounded process; merus slightly expanded, anterior margin crenulate, with nine robust setae, posterior margin convex and with six robust setae; carpus as long as merus, anterior margin crenulate and with four groups of robust setae, posterior margin crenulate with four groups of robust setae; propodus longer than merus and carpus, anterior margin slightly crenulate with 13 robust setae, posterior margin slightly crenulate with 16 robust setae; dactylus slender with subapical robust setae. ***Pereopod 7*** (Fig. 10, P7) not sexually dimorphic, coxa reduced, ventral margin with minute setae; basis expanded without lateral sulcus, posterodistal lobe present, anterior margin with ten robust setae, posterior margin serrate with minute setae, ischium shortest with posterior process; merus as long as carpus, anterior margin slightly crenulate with ten robust setae, posterior margin with posteroventral lobe and five robust setae; carpus oblong; anterior margin slightly crenulate with eight robust setae, posterior margin crenulate with six robust setae; propodus slender, longer than merus and carpus, 1.17× as long as carpus anterior margin with 13 robust setae, posterior margin with eight robust setae; dactylus slender, apically acute without robust setae.

***Pleon. Pleopods 1–3*** well developed, biramous; ***Pleopod 1*** (Fig. 11, PL1) peduncle longer than rami, without marginal robust setae; inner ramus as long as outer ramus, with 15 articles; outer ramus with 14 articles. ***Pleopod 2*** (Fig. 11, PL2) peduncle slightly longer than rami, with marginal robust setae on posterior margin; inner ramus as long as outer ramus, with 12 articles; outer ramus with 12 articles. ***Pleopod 3*** (Fig. 11, PL3) peduncle longer than rami, posterodistal margin with 3 robust setae; inner ramus longer than outer ramus. with 11 articles; outer ramus with ten articles. ***Epimera 1–3*** posterior margin slightly serrate, posteroventral corners of epimera 2 and 3 reduced. ***Uropod 1*** (Fig. 11, U1) peduncle 1.5× longer than rami, with seven robust setae in two rows, without distolateral robust setae; inner ramus subequal in length to outer ramus, with four marginal robust setae and three apical robust setae; outer ramus without marginal robust setae and with three apical robust setae. ***Uropod 2*** (Fig. 11, U2) peduncle 1.3× longer than rami, with four marginal robust setae; inner ramus with four marginal robust setae and 3 apical robust setae; outer ramus with one or two marginal robust setae and three apical robust setae. ***Uropod 3*** (Fig. 11, U3) peduncle subequal in length to ramus, with three robust setae; ramus slender, more than 3.5× longer than broad, with one marginal robust seta and four apical robust setae. ***Telson*** (Fig. 11, T) longer than broad, apically incised, with three marginal robust setae and three apical robust setae per lobe; dorsal midline entire.

**Male** (minor form). Based on paratype, male 7.9 mm. THNHM-lv-20871.

**Head. *Antenna 2*** (Fig. 12, A2) peduncle not incrassate, peduncle slender.

**Pereon. *Gnathopod 1*** (Fig. 12, G1); basis slightly expanded posteriorly, anterior margin with two robust setae, posterior margin with two robust setae; carpus 1.4× longer than propodus; propodus anterior margin with two groups of robust setae; dactylus subequal to palm. ***Gnathopod 2*** (Fig. 12, G2) coxa ventral margin with ten robust setae; basis anterior margin slightly concave with two robust setae, posterior margin expanded with four robust setae; ischium subrectangular, anterior margin with lobe; merus longer than carpus, convex on posterior margin; carpus triangular; reduced; enclosed by merus and propodus, posterior lobe absent; propodus subovate, palm acute reaching 45% along posterior margin, 1.4× as long as wide, palmar margin slightly convex with six robust setae; dactylus longer than palm and fitting in facial groove of propodus, attenuated distally.

**Female** (sexually dimorphic characters) based on allotype type, female 7.8 mm. THNHM-lv-20870.

***Pereon. Antenna 2*** (Fig. 12, A2♀) peduncle slender. ***Gnathopod 1*** (Fig. 12, G1♀) parachelate; posterior margin of merus, carpus and propodus without lobe covered in palmate setae; basis slightly expanded with posterodistal rounded process, anterior margin with six robust setae, posterior margin with three robust setae; ischium shortest, anterior margin with rounded lobe; merus triangular, posterior margin with six robust setae; carpus subtriangular, anterior margin straight with four robust setae, posterior margin convex with six robust setae in two groups, propodus subrectangular, palm acute, anterior margin with nine robust setae, posterior margin with 11 robust setae; dactylus much longer than palm, posterior margin with three robust setae. ***Gnathopod 2*** (Fig. 12, G2♀) mitten-shaped, coxa posterior margin with acute process, ventral margin with minute setae; basis expanded, anterior margin convex with minute setae; ischium subrectangular, anterior margin with round lobe, posterior margin with four robust setae; merus, carpus, and propodus each with lobe covered in palmate setae; merus posterior lobe well developed with seven robust setae; carpus subtriangular; propodus subovate, palm obtuse, laterodistal corner with six robust setae in one line; dactylus slender, not longer than palm.

##### Distribution.

Thailand. Klong Mai, Bang Pu, Samut Prakan.

##### Etymology.

Named for the freshwater habitat where the species was collected.

##### Remarks.

*Platorchestiaaquaticus* sp. nov. is the first species of *Platorchestia* recorded from Southeast Asia. The genus *Platorchestia* is classified as supra-littoral and terrestrial and is sexually dimorphic in antenna 2 and gnathopods 1 and 2. Males present two different forms in the population. The major form of *P.aquaticus*, based on the male holotype, appears similar to *P.pacifica* (Miyamoto & Morino, 2004), *P.paraplatensis* (Serejo & Lowry, 2008), and *P.smithi* (Lowry, 2012) in the following characteristics: 1) mandible left lacinia mobilis 5 dentate, 2) coxae 3 and 4 as wide as deep, 3) gnathopod 1 dactylus slightly short or subequal to palm, 4) gnathopod 1 propodus subtriangular, palm transverse, 5) Uropod 3 peduncle with two or three robust setae, 6) telson longer than broad. However, *Platorchestiaaquaticus* sp. nov. may be distinguished from other closely related species as follows: 1) gnathopod 1 with rudimentary cusps on dactylus (*P.pacifica* and *P.paraplatensis* gnathopod 1 with distinct cusp on dactylus), 2) propodus of gnathopod 2 without notch on palmar margin, 3) uropod 1 peduncle with nine or ten robust setae in two rows, 4) uropod 3 ramus with single robust marginal seta, 5) telson with three robust marginal setae and three robust apical setae per lobe.

### ﻿Key to species of the *Floresorchestia* in Southeast Asia and neighboring regions (modified from Suklom et al. 2022)

**Table:** 

1	Gnathopod 1 carpus subequal or ? (1.7×) to propodus	**2**
–	Gnathopod 1 carpus > 1.7× than propodus	***F.thienemanni* (Schellenberg, 1931)**
2(1)	Gnathopod 1 carpus significantly > 1.2–1.5× than propodus	**3**
–	Gnathopod 1 carpus subequal in length to propodus	**5**
3(2)	Gnathopod 1 carpus 1.2–1.3× than propodus	**4**
–	Gnathopod 1 carpus significantly > 1.5× than propodus	**10**
4(2)	Telson approximately as long as broad; antenna 2 longer than head and first 3 pereonites	***F.seringat* Lowry & Springthorpe, 2015**
–	Telson longer than broad; antenna 2 shorter than head and first 3 pereonites	***F.kongsemae* Suklom et al., 2021**
5(3)	Gnathopod 2 propodus 1.3–1.4× as long as wide	**6**
–	Gnathopod 2 propodus 1.5–1.8× as long as wide	**9**
6(5)	Uropod 1 peduncle without robust setae	***F.malayensis* (Tattersall, 1922)**
–	Uropod 1 peduncle with 4–6 robust setae	**6**
7(5)	Left lacinia mobilis 5-dentate	***F.pongrati* Suklom et al., 2022**
–	Left lacinia mobilis 4-dentate	**8**
8(7)	Uropod 3 peduncle with 2 robust setae; telsonic lobe with 3 robust setae	***F.trisetosa* sp. nov.**
–	Uropod 3 peduncle with 4 robust setae; telsonic lobe with 4 robust setae	***F.amphawaensis* Suklom et al., 2022**
9(5)	Left lacinia mobilis 5-dentate; uropod 1 peduncle without marginal setae; uropod 3 ramus without marginal robust setae	***F.yehyuensis* Miyamoto & Morino, 2008**
–	Left lacinia mobilis 4-dentate; uropod 1 peduncle bearing more than 6 robust setae; uropod 3 ramus with 1 marginal seta	***F.buraphana* Wongkamhaeng et al., 2016**
10(3)	Epimera 2 and 3 with slits	**11**
–	Epimera 1–3 with slit	***F.laurenae* Lowry & Springthorpe, 2015**
–	Epimera 1–3 without slits	***F.xueli* Tong & Hou, 2021**
11(9)	Gnathopod 2 propodus 1.4× as long as wide	**12**
–	Gnathopod 2 propodus 1.5–1.6× as long as wide	**13**
12(11)	Telson broader than long; Gnathopod palm without protuberance near dactylar hinge; Uropod 3 ramus without marginal robust setae	***F.hanoiensis* Hou & Li, 2003**
–	Telson longer than broad; Gnathopod palm with rounded protuberance near dactylar hinge; Uropod 3 ramus with marginal robust setae	***F.floresiana* (Weber, 1892)**
13(11)	Gnathopod palm reaching 40–50% of propodus	**14**
–	Gnathopod palm reaching 30–40% of propodus	***F.boonyanusithii* Wongkamhaeng et al., 2016**
14(13)	Uropod 2 outer ramus with 2 marginal setae	***F.anpingensis* Miyamoto & Morino, 2008**
–	Uropod 2 outer ramus with 2 marginal setae	***F.oluanpi* Lowry & Springthorpe, 2015**

## ﻿Discussion

The genus *Floresorchestia* occupies several ecological types, including marsh hoppers, field hoppers, beach hoppers, riparian hoppers, and forest hoppers (Lowry and Myers 2019). In Thailand, species of *Floresorchestia* are either field hoppers (*F.buraphana*, *F.boonyanusithii*, and *F.kongsemae*) or marsh hoppers (*F.amphawaensis* and *F.pongrati*) (Suklom et al. 2021, 2022). *Floresorchestiatrisetosa* sp. nov. is the first record of riparian hopper to be recorded in Thailand. Another riparian hopper reported from Southeast Asia is *F.thienemanni*, which is present near a waterfall among the stand of aroid *Colocasia* in central Java, Indonesia, at 1,400 m altitude. The new species *F.trisetosa* sp. nov. occupies the edge of a small creek in Muang Trat District, Trat at 0 m a.s.l.; therefore, the distribution of *Floresorchestia* is not affected by altitude.

The subfamily Floresorchestiinae is characterized by vertical slits on the ventral margin of epimera 1–3 (Myers and Lowry 2020), but *Floresorchestiaxueli* lacks this character. Therefore, the taxonomic status of *F.xueli* should be revised.

Morino (2024) described the variability of certain characteristics of *Platorchestia* and *Dermaorchestia* from the coast of Japan. This variability included 12 characters. The new species *Platorchestiaaquaticus* sp. nov. presents variations in the following characters: 1) article 3 of antenna 1 with four marginal robust setae; 2) antenna 2 peduncle slightly incrassate; 3) gnathopod 1 without cusps on dactylus; 4) ratio of propodus to carpus of gnathopod 1 ~ 0.67; 5) gnathopod 2 posterior margin with sharp cusps; 6) gnathopod 2 palm with smooth margins; 7) without robust setae on posterior margin of gnathopod 2; 8) ratio of carpus length to width ~ 1.67; 9) pereopod 6 posterior lobe of coxa without protrusion; 10) pereopod 7 carpus not incrassate; 11) the number of robust setae on the outer margins of pleopods 2 and 3 with three and two robust setae, respectively; 12) six robust setae on the left lobe of telson.

**Table 1.** A summary of the diagnostic characteristics that serve to distinguish closely related *Platorchestia* species (P = peduncle, In = inner ramus, Out = outer ramus, M = marginal, A = apical).

Species	A2 peduncle	G1 dactylus	G1 carpus	G2 palm margin	G2 posterior notch	Pereopod 6 coxa	Pereopod 7 carpus	Uropod 1 inner ramus	Uropod 2	Uropod 3	Telson
*Platorchestiaano* Lowry & Bopiah, 2013	5^th^ longer than 4^th^	Subequal in length to palm, cuspidactylate	2× longer than propodus	rounded	–	Not protruded	slender	3 robust setae	P 7–8	P 3	7 per lobe
									In 4	M 2	
									Out 2	A 4–5	
*Platorchestiacrassicornis* (Costa, 1867)	–	Longer than palm, -	1.5× longer than propodus	2 strong processes	–	–	Not strong	–	–	–	8 per lobe
*Platorchestiaexter* Myers & Lowry, 2023	5^th^ longer than 4^th^	Shorter than palm, cuspidactylate	Less than 3× of width	Slightly mid notch		Distinctly protruded	very enlarged	7 robust setae	P 7–8	P 1	3–5 per lobe
									In 2	M 3	
									Out 1	A3–4	
*Platorchestiagriffithsi* Myers & Lowry, 2023	5^th^ longer than 4^th^	Much shorter than palm	Over 1.5× of propodus	with subdistal notch	–	Not protruded	Incrassate, subovoid	6 robust setae	P 7–10	P 3	3–5 per lobe
									In 1 row	M 1	
									Out 1	A 3	
*Platorchestiamunmui* Jo, 1988	5^th^ 1.4× as long as 4^th^	Shorter than palm	–	Notch near posterior	acute	Not protruded	incrassate	+	–	–	8 per lobe
*Platorchestianegevensis* Myers & Lowry, 2023	5^th^ longer than 4^th^	Shorter than palm, cuspidactylate	–	Nearly straight	–	Not protruded	slender	8 robust setae	P 8–9	P 3	5–7 per lobe
									In 6	M 2	
									Out 2	A 3	
*Platorchestiaoliveirae* Myers & Lowry, 2023	5^th^ longer than 4^th^	Overlapping palm	–	strong mid palmar notch	–	Not protruded	Weakly expanded	7 robust setae	P 7–10	P 1	5–6 per lobe
									In 2	M 2–3	
									Out -	A 3–4	
*Platorchestiapachypus* (Derzhavin, 1937)	–	Shorter than palm, cuspidactylate	–	rounded	–	Not protruded	Distinctly incrassate	1 subapical 2 apical	–	–	+
*Platorchestiapacifica* Miyamoto & Morino, 2004	5^th^ 1.4× as long as 4^th^	Shorter than palm, cuspidactylate	0.6× as long as propodus	with subdistal notch	acute	Distinctly protruded	weakly incrassate	7 robust setae	P 6	P 4	5 per lobe
									In 4	M 3	
									Out 1	A 3	
*Platorchestiaparaplatensis* Serejo & Lowry, 2008	–	Shorter than palm, cuspidactylate	–	Well-developed mid-palmar notch	obtuse	Distinctly protruded	incrassate	7 robust setae	P 7–10	P 2–3	3–5 per lobe
									In 5	M 2	
									Out 3	A 4–5	
*Platorchestiaplatensis* (Krøyer, 1845)	5^th^ 1.4× as long as 4^th^	Weakly overlapping palm	–	with subdistal notch	acute	Distinctly protruded	incrassate subovate	7 robust setae	P 8	P 2–3	3–5 per lobe
									In 2	M 0–2	
									Out 2	A 3–4	
*Platorchestiasmithi* Lowry, 2012	5 subequal than article 4	subequal in length to palm	–	Weakly notch	smooth	Distinctly protruded	slender	11 robust setae	P 4	P 3	3–6 per lobe
									In 1	M 2	
									Out 1	A 5	
*Platorchestiaaquaticus* sp. nov.	5^th^ 1.7× as long as 4^th^	Shorter than palm, cuspidactylate	1.3× longer than propodus	rounded	smooth	Not protruded	slender	4 robust setae	P 6	P 3	6 per lobe
									In 4	M 1	
									Out 1–2	A 4	

**Figure 13.**
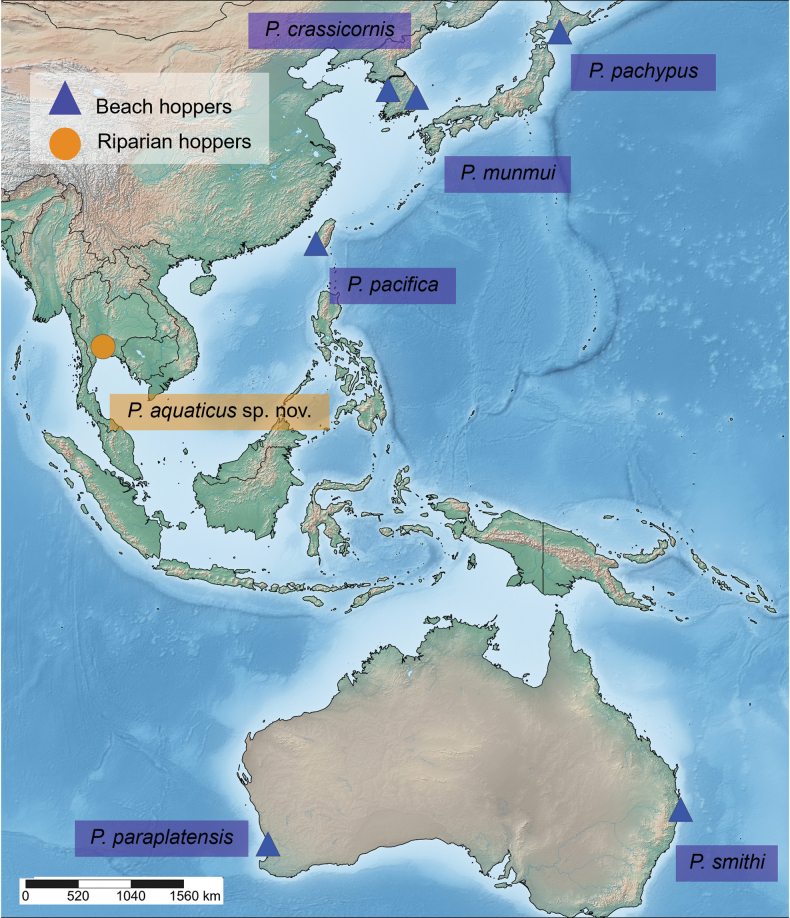
Ecological groups of *Platorchestia* species in the Indo-Pacific region.

Previously, *Platorchestia* species in the Indo-Pacific were classified as beach hoppers, primarily residing among algal debris on upper marine shores and occasionally found in estuaries and mangrove habitats (Lowry and Myers 2019, 2022; Myers and Lowry 2023). Significantly, *Platorchestiaaquaticus* sp. nov. was found on the edge of the Mai freshwater canal, which runs parallel to Sukhumvit Road and is not directly connected to the coast (Fig. 13).

*Platorchestiaparaplatensis* and *P.griffithsi* are two extant species present on the margin of the Swan River, Western Australia and Knysna Lagoon, South Africa, respectively, which are considered to be brackish water. *Platorchestianegevensis* was found near springs and wells in the Negev desert, Israel (Morino and Ortal 1995). Due to their habitat on the edge of water bodies, *P.negevensis* and *P.aquaticus* sp. nov. are considered to be riparian hoppers. Two hypotheses of amphipod invasion were proposed by Herbst and Dimentman (1983) as follows: the marine origin that penetrated into inland waters and the sea level decreased and left the amphipods along the regression (Herbst and Dimentman 1983). The second hypothesis possibly explains the appearance of *P.aquaticus* sp. nov. and *P.negevensis*, which may have settled down in their (type) localities after the sea level decreased.

## Supplementary Material

XML Treatment for Floresorchestia

XML Treatment for Floresorchestia trisetosa

XML Treatment for Platorchestia

XML Treatment for Platorchestia aquaticus

## ﻿Additional information

### Author ORCIDs

Tosaphol Saetung Keetapithchayakul https://orcid.org/0000-0001-7565-4701

Azman Abdul Rahim https://orcid.org/0000-0002-8964-6638

Koraon Wongkamhaeng https://orcid.org/0000-0001-7671-8869

### Data availability

All of the data that support the findings of this study are available in the main text.
